# Transarterial (Chemo-)Embolization and Lipiodolization for Hepatic Haemangioma

**DOI:** 10.1007/s00270-019-02169-x

**Published:** 2019-02-19

**Authors:** Alicia Furumaya, Belle V. van Rosmalen, R. Bart Takkenberg, Otto M. van Delden, Cornelis H. C. Dejong, Joanne Verheij, Thomas M. van Gulik

**Affiliations:** 1 Department of Surgery, Amsterdam UMC, University of Amsterdam, Amsterdam, The Netherlands; 2 Department of Gastroenterology and Hepatology, Amsterdam UMC, University of Amsterdam, Amsterdam, The Netherlands; 3 Department of Interventional Radiology, Amsterdam UMC, University of Amsterdam, Amsterdam, The Netherlands; 4 Department of Surgery, Maastricht University Medical Centre, Maastricht, The Netherlands; 5 Department of Surgery, Uniklinikum Aachen, Aachen, Germany; 6 Department of Pathology, Amsterdam UMC, University of Amsterdam, Amsterdam, The Netherlands; 7 Department of Surgery, Amsterdam UMC, University of Amsterdam, Meibergdreef 9, Room G4-108, 1105AZ Amsterdam, The Netherlands

**Keywords:** Hepatic haemangioma, Benign liver tumour, Lipidolization, Transarterial chemoembolization, Embolization, Interventional radiology

## Abstract

**Background:**

Transarterial (chemo-)embolization/lipiodolization (TAE/TAL) might be an attractive minimally invasive alternative to surgery in the treatment of symptomatic hepatic haemangioma. This review assesses the efficacy and safety of TAE/TAL as primary treatment for symptomatic hepatic haemangioma.

**Methods:**

A systematic search of the literature was performed by two reviewers following the PRISMA guidelines. Cohort studies and case reports were identified; outcomes of cohort studies were reported. The primary efficacy outcome was tumour size before and after TAE/TAL. Improvement of symptoms and quality of life (QoL) were secondary outcomes; the primary safety outcome was complications. The Downs and Black statement was used for quality assessment.

**Results:**

Eighteen cohort studies were identified, including 1284 patients. TAE/TAL led to a decrease in tumour size in 1100/1223 (89.9%) patients and to improvement or disappearance of symptoms in 1080/1096 (98.5%) patients. A significant decrease in tumour size from 9.79 ± 0.79 cm to 4.00 ± 1.36 cm (*p* < 0.001) was shown. Grade 3 complications occurred in 37/1284 (2.9%) patients. Surgical treatment was necessary in 35/1284 (2.7%) of patients.

**Conclusion:**

TAE/TAL appears to be a promising and safe treatment to decrease tumour size of hepatic haemangioma. The technique might also provide partial relief of symptoms, although no randomized clinical trials or prospective studies using validated QoL questionnaires are available. TAE/TAL may be considered as a viable alternative to resection.

**Electronic supplementary material:**

The online version of this article (10.1007/s00270-019-02169-x) contains supplementary material, which is available to authorized users.

## Introduction

Haemangioma, occurring predominantly in middle-aged women, is the most common benign liver tumour [1–5]. The reported incidence varies from 0.4 to 20% in the general population [1, 2, 6, 7]. In most cases, no symptoms are reported and the tumour is discovered incidentally on imaging for unrelated pathologies [8–13]. No treatment or follow-up imaging is required in such cases [11, 14–20].

Treatment indications are symptomatic (i.e. abdominal pain, nausea and early satiety) haemangioma, progressive growth and high risk of bleeding. Frequently, an open or laparoscopic surgical approach is used for resection of symptomatic haemangioma [10, 18, 21–25]. Common complications of surgery are blood loss, bile leakage, ileus and wound infection with a reported morbidity of 13–21% and a mortality of 0–2% [8, 26–30].

Haemangiomas are composed of endothelial cells from the hepatic artery [8, 12, 31], suggesting a place for vessel occlusive therapies such as transarterial embolization (TAE). Chemotherapeutic agents may be added to TAE with the intention to achieve additional volume reduction through the prevention of blood vessel (re)growth; this is called transarterial chemoembolization (TACE). Chemotherapeutic agents can also be administered without vessel occlusive agents, for example, in combination with ethiodized oil (lipiodolization) [32, 33]. TAE has been used as a treatment for hepatocellular adenoma, with promising results [34]. In recent years, transarterial (chemo-)embolization and lipiodolization (TAE/TAL) have been suggested to be effective in the treatment of hepatic haemangioma [13, 31, 35], either preoperatively to reduce intraoperative blood loss [13] or as definitive treatment [8, 31, 35]. Due to the non-invasive nature of the procedure, cosmetic outcomes are favourable. However, TAE/TAL does entail exposure to radiation. No comprehensive evaluation of TAE/TAL in the literature has been performed to our knowledge.

The aim of this review is to assess the safety and efficacy of TAE/TAL as definitive treatment for haemangioma of the liver.

## Methods

### Study Identification

The databases MEDLINE (PubMed) and Embase were systematically searched with the aid of a clinical librarian (F.v.E.). Articles were screened by title and abstract by two independent reviewers (A.F. and B.V.v.R); inconsistent judgement was resolved by consensus. The PRISMA guidelines were followed throughout the entire process [36]. The search strategy and full study protocol are included in supplementary file 1.

### Inclusion and Exclusion Criteria

Reviews, studies not in English, French or German, studies including patients under the age of eighteen or patients with extra-hepatic haemangioma, concomitant malignancies, inherited or syndromal disease or ruptured haemangioma were excluded. If full texts were unavailable, they were purchased. Studies reporting on patients with hepatic haemangioma who had undergone TAE/TAL were included. Cohort studies were included, with no limit of a minimal number of patients; data of case reports were reported separately in tables S3–S6.

### Data Collection and Definitions

Data were extracted using a standardized data extraction form. The following characteristics were collected: study design, age, sex, number of patients included, number of tumours per patient and follow-up duration, embolic agent, number of procedures, prophylactic and supportive care and imaging modalities used before and after treatment. Technical failure, as defined in the supplementary appendix, was also recorded. Indications for treatment were recorded. (Please note that the presence of symptoms before and after treatment was not the same parameter as indication for treatment.) Symptoms were recorded as the reason for treatment only if explicitly stated. Tumour size was recorded as the reason for treatment only if explicitly stated (i.e. because of damage to surrounding structures, vena cava inferior syndrome or dietary problems caused by pressure on the stomach).

The aim of this review was to determine the efficacy and safety of TAE/TAL treatment. The primary outcome measure for efficacy was tumour size. The following outcome parameters were assessed: tumour size before and after the procedure (in cm), changes in tumour size according to the RECIST criteria [37], number of TAE/TAL sessions, number of patients undergoing surgery after TAE/TAL and reason to proceed to surgery. The RECIST criteria are commonly used for malignant liver tumours but were applied for benign disease. If available, data on volumetric analysis were also extracted [38].

Secondary outcomes regarding efficacy of TAE/TAL were (relief of) symptoms and quality of life (QoL). Therefore, the presence and type of symptoms at presentation and after TAE/TAL were recorded. If the extent of symptom relief was not reported, this was recorded as partial relief to prevent an overestimation of treatment effect.

Safety was measured by recording complications according to the Cardiovascular and Interventional Radiological Society of Europe (CIRSE) classification [39]. Finally, type of complication and TAE-/TACE-related mortality and overall mortality were reported.

### Quality Assessment

The Downs and Black criteria were used to assess methodological quality [40]. Criteria of the Downs and Black tool are provided in Table S1.

### Statistical Analysis

Percentages calculated for baseline characteristics may also include data of patients not undergoing TAE/TAL if a study did not subdivide their baseline data according to treatment method. For the outcome measures, only data of patients who underwent TAE/TAL were analysed. If data were not reported separately for patients undergoing TAE/TAL, all patients were excluded for analysis. This led to varying denominators for each outcome measure. Patients with failed embolization/lipiodolization were included in outcome analyses, following the intention-to-treat principle.

Studies reporting their data as a change in mean tumour diameter including standard deviation (SD) were analysed by weighted average, and a paired *t* test was performed.

## Results

**Fig. 1:**
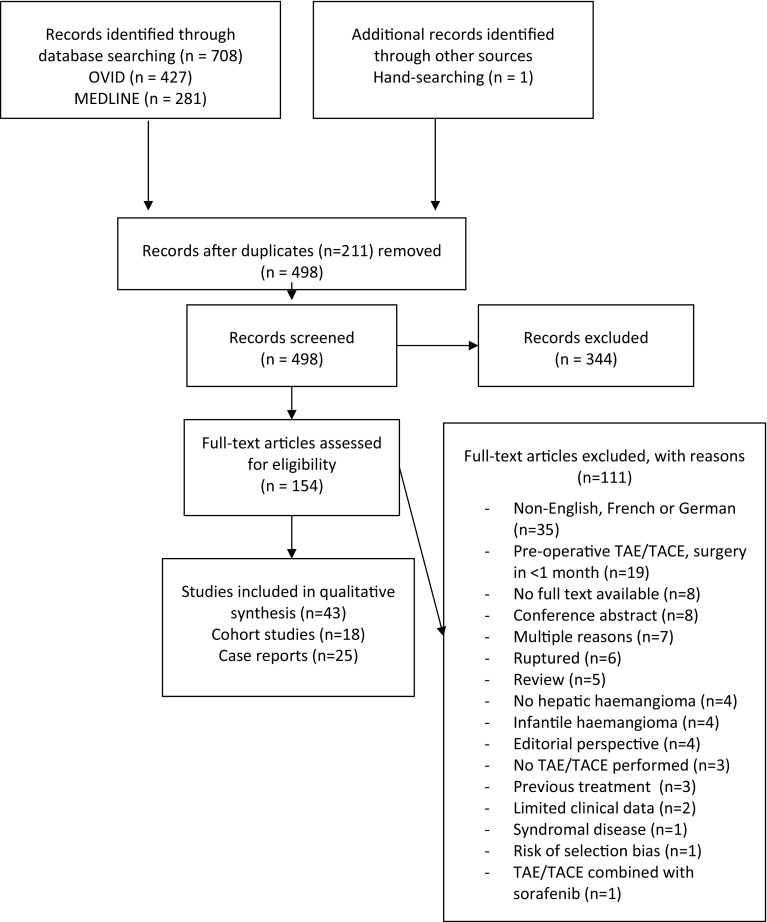
PRISMA flow chart of the study selection process

The original search yielded 705 articles; one other article was identified by hand-searching. Then, one copy of the articles that were identified twice, in the MEDLINE as well as in the OVID search (duplicates),was removed. After removal of these duplicates, 496 articles underwent title and abstract screening. Subsequently, 154 articles were assessed by full text. Finally, a total of 18 cohort studies were eligible for inclusion [8, 16, 31, 38, 41–52], five of which had a prospective design [31, 49, 51–53]. The full study selection process is shown in Fig. 1.

### Quality Assessment

Supplementary table 1 shows the full critical appraisal of each individual study. The studies by Sun et al. [50], Ji et al. [43] and Li et al. scored highest on the assessment with the Downs and Black: 15, 14 and 13 points, respectively [31]. The studies by Cao et al. [41], Martin et al. [46], Li et al. had the lowest scores: 6, 6 and 4 points, respectively [44].

### Baseline Characteristics

A total of eighteen articles including 1531 patients met the eligibility criteria. Baseline characteristics of these patients are shown in Table 1. Of these patients, 1284 underwent TAE/TAL. The majority was female, 981/1522 (64.5%), and the weighted mean age was 43.5 years. In 302/1320 (22.9%) patients, multiple haemangiomas were identified. The number of tumours in the left, right and both lobes was 81/428 (18.9%), 256/428 (59.8%) and 91/428 (21.3%) tumours, respectively. Comorbidity is reported in table S2. Patients were followed for a weighted mean of 46.8 months.

**Table 1:** Baseline characteristics of patients included in cohort studies

	Total/embolized	Solitary/multiple	Left/right/both	Age (years)	Female/male	Mean follow-up (months)
Akhlaghpoor et al. [54]	23/23	20/3, T: 29	9/20/0^i^	46.7	25/4^i^	7.5
Bozkaya et al. [8]	26/26	T: 32	4/24/4	49.8	21/5	7.4
Cao et al. [41]	14/14	NA	NA	44.0	7/7	21.0^f^
Farges et al. [16]	163/5	108/55^a^	NA	48.1^a^	123/40^a^	92.0^a^
Firouznia et al. [42]	20/20	20/5, T: 25	8/17/0	46.8	16/4	6.0
Ji et al. [43]	15/15	10/5^b^	2/8/5	43.5	9/6	7.0
Kirnap et al. [53]	17/17	6/11	2/7/8	46.4	10/7	14.5
Li et al. [44]	75/10	56/19^a^	14/53/12^a^	37.4^a^	45/30^a^	6.0
Li et al. [31]	836/836	720/116, T: 1120	NA	42.8	535/301	52.8
Liu et al. [45]	55/55^h^	NA	NA	43.1	38/17	60.0
Martin et al. [46]	7/7	2/5	NA	40.4	5/2	38.8^e^
Ouyang et al. [47]	30/21	8/22	4/13/13	43.0^a^	25/5^a^	NA
Özden et al. [38]	25/25	9/16^c^	NA	47.6	21/4	14.0^g^
Reading et al. [48]	24/9	NA	NA	45.0	6/3	24.0^ag^
Srivastava et al. [49]	8/8	7/1, T: 9	1/5/2	47.8	3/5	9.0^f^
Sun et al. [50]	27/27	10/17	1/13/13	47.7	21/6	9.6
Wang et al. [51]	68/68	42/26	20/35/12	41.0	45/23	13.5^f^
Zeng et al. [52]	98/98	NA	16/61/21	41.6	26/72	12.0
Total	1531/1284	1018/302, T: 1215	81/256/91	43.5^d^	981/541	46.8^d^

Total/embolized = number of patients with haemangioma/number of patients undergoing TAE/TAL for haemangioma, Solitary/multiple = number of patients with solitary haemangioma/number of patients with multiple haemangioma, T = total number of haemangioma, Left/right/both = number of haemangioma in the left liver lobe/number of haemangioma in the right liver lobe/number of haemangioma in both liver lobes, Age = mean age in years, NA = not available

^a^Reported for all patients with haemangioma, not only patients undergoing TAE/TAL

^b^Unclear method of reporting: multiple haemangiomas in one lobe are counted as single haemangioma

^c^Includes twelve patients with > 3 lesions

^d^Weighted average

^e^Two patients lost to follow-up

^f^Follow-up reported as a range converted to a mean

^g^Reported as a median

^h^Eight patients excluded due to loss to follow-up

^i^Reported for the number of lesions, not the number of patients

### Indications for Treatment

The indication for treatment was clearly stated for 231/1284 patients (18.0%) [8, 16, 38, 41–43, 45, 48–50, 53]. The main treatment indication was tumour size (104/231 patients; 45.0%). Another reason for treatment was incapacitating symptoms (83/231 patients; 35.9%). Multiple reasons for treatment, present in 39/231 patients (16.9%), always included symptoms. Three studies stated that the indication for treatment was either symptoms, fear of future bleeding, patients’ desire for treatment or tumour size, but did not specify how many patients underwent treatment for each indication [51, 52, 54]. Details are tabulated in Table 2.

**Table 2:** Reason for treatment and outcomes in cohort studies

	Indications for TAE/TAL	Reasons (not) to perform additional procedures	Tumour size reduction	Follow-up	Symptoms	S.I	Complications
Akhlaghpoor et al. [54]	NA	Elective: reason not stated	R: 29	7.5	PD: 23	NA	Grade 1 (13): non-target embolization Grade 1 (6): PES
Bozkaya et al. [8]	AS: 24 E: 2	Elective (26): patients unfit for surgery	Ø 9.72–7.63 S: 26	7.4	SO: 26	I: 26	Grade 3 (1): ischaemic cholecystitis Grade 1 (26): PES
Cao et al. [41]	L: 14	Elective: unresectable tumour	R:8, > 50% reduction	1	NA	D: 14	Grade 1 (11): PES
Farges et al. [16]	AS: 16^a^	Elective: directed at intratumoural arterial fistula (1), reason not stated (4)	R: 7, G: 9^a^	NA	PD: 87^a^	D: 1 P: 4	None
Firouznia et al. [42]	AS: 15 E: 5	Elective: reason not stated	S: 20, Ø 9.70–8.90	6	NA	I: 20	Grade 2 (1): PES
Ji et al. [43]	AS: 3 E: 4 M: 8	Elective: refusal to surgery. RFA after TAE/TAL	R: 15, Ø 13.0–7.1	1	PD: 15	D: 10 I: 1 NA: 4	Grade 3 (8): postprocedural pain
Kirnap et al. [53]	M: 17	Elective: reason not stated	R: 17, Ø 14.7–7.6, mean volume 3716 cm^3^ to 746 cm^3^	12	PD: 5 B: 11 O: 1	I: 17	Grade 3 (1): low haemoglobin Grade 1 (7): PES
Li et al. [44]	NA	Elective: experimental	S: 10	6.0		NA	Grade 3: lasting pain^e^
Li et al. [31]	NA	Elective: patients choice	R: 836, Ø 9.6–3.6	NA	SO: 836	I: 836	Grade 3 (2): hepatic abscess Grade 1: PES^e^
Liu et al. [45]	L: 55	Elective (24): reason not stated Pre-operative (31): enlarging tumour (29) or severe complication (2)	S: 19 G: 34	NA	PD: 8 B: 20 M: 2 A: 25	NA	Grade 3 (2): biloma Grade 3 (2): hepatic abscess
Martin et al. [46]	NA	Elective: reason not stated	R:2, S: 2^b^	21^b^	PD: 3 B: 2 ME: 1 A: 1	D: 1 I: 2 P: 2 NA: 2^b^	Grade 1 (3): postembolization pain
Ouyang et al. [47]	NA	Elective: directed at arteriovenous shunt	NA	NA	PD: 6 A: 24^a^	NA	NA
Özden et al. [38]	AS: 15 E: 3 M: 7	Elective: reason not stated	Median volume 466 cm^3^ to 108 cm^3^	8^d^	A: 3	I: 17 P: 5 NA: 3	Grade 3 (1): transient allergic rash Grade 2 (3): PES Grade 1 (22): PES
Reading et al. [48]	AS: 7 H: 1 O: 1	Elective: reason not stated	R:1, S:6^c^	NA	PD: 3 M: 3 A; 1 ME: 1 O: 1	D:1 I: 1 P: 5	Grade 3 (2): hepatic abscesses Grade 3 (6): PES
Srivastava et al. [49]	M: 7	Elective: experimental Pre-operative (1): persistent symptoms	R: 1, S: 4, Ø 9.28–8.62	9	PD: 6 O: 2	D: 8	Grade 1 (8): PES
Sun et al. [50]	AS: 3 E: 21 O: 3	Elective: reason not stated. Pre-operative (1): surgery possible after TAE/TAL, 3 weeks post-TAE/TAL	R: 27, Ø 11.24–7.60	6	PD: 2 O: 1 A: 24	I: 4 NA: 23	Grade 3 (12): mild fever Grade 1 (6): PES
Wang et al. [51]	NA	Elective: experimental	S: 2, R: 66, 30 of which with > 50% reduction	NA	SO: 47 A: 21	I: 68	Grade 1: haematoma at puncture site Grade 1: PES^e^
Zeng et al. [52]	NA	Elective: patient’s choice. Pre-operative (2): continued pain	R: 98, Ø 9.70–3.00	12	M: 53 A: 45	D: 46 I: 7 NA: 45	Grade 1 (83): PES
Total	AS: 83 L: 69 E: 35 M: 39 O: 4 H: 1	Pre-operative (35): enlarging tumour (29), unfit for surgery (26), continued symptoms (3), severe complication (2) or surgery possible (1)	R: 1100 S: 89 G: 34	8,6^f^	SO: 909 A: 120 PD: 65 M: 56 B: 33 ME: 4 O: 5	D: 81 I: 999 P: 16	Grade 3: 37 Grade 2: 4 Grade 1: 185

Follow-up = timing of follow-up imaging in months, Symptoms = symptoms before TAE/TAL, SI = symptomatic improvement, AS = in order to alleviate symptoms, E = enlarging tumour, L = large tumour, H = haemorrhage, high bleeding risk, O = other, Ø = mean diameter in cm before TAE/TAL—mean diameter after TAE/TAL. RECIST Criteria: CD = Complete disappearance of the tumour, R = reduction (> 30%) decrease in tumour size, S = stable (< 30%) decrease and (< 20%) increase in tumour size, G = growth (> 20%) increase in tumour size., PD = pain or discomfort, B = bloating or abdominal distension, M = multiple, ME = mass effect including dyspepsia and palpable masses, SO = symptoms not otherwise specified, A = asymptomatic, D = disappearance, I = improvement, P = persistent/recurrent/stable symptoms, PES = postembolization syndrome, NA = not available

^a^Reported for all patients with haemangioma, not only patients undergoing TAE/TAL. Excluded from analysis

^b^Two patients lost to follow-up, one patient no imaging follow-up

^c^Includes patient treated with open cannulation

^d^Median

^e^Not reported how many patient suffered from these complications

^f^Weighted average

### Diagnostic and Treatment Methods

Table 3 shows an overview of diagnostic and treatment methods. Mostly, ultrasound, computed tomography and magnetic resonance imaging were applied. Biopsy was occasionally used in case of unsure diagnosis or if the study was conducted before widespread availability of CT and MRI [16, 31, 38, 48, 51]. Lipiodolization was performed with either pingyangmycin or bleomycin [8, 31, 38, 45, 47, 50, 52–54]. TAE/TACE procedures usually included the use of gelfoam [41, 43, 44, 46, 48, 49]. Use of prophylactic antibiotic therapy was reported in six studies [8, 38, 48, 49, 53, 54]; supportive treatment, usually analgesia and antiemetics, was used in twelve studies [8, 31, 38, 41, 43, 45, 48–51, 53, 54]. One study used glutathione for reasons not mentioned by the authors [31].

**Table 3:** Diagnostic and treatment methods used in cohort studies

	Diagnostic methods before TAE/TAL	Diagnostic methods after TAE/TAL	TAE/TAL material	Nr. TAE/TAL	Pharmacotherapeutics
Akhlaghpoor et al. [54]	US, CT or MRI	CT	Bleomycin (30–45 IU in 5 cc saline) and lipiodol (7–15 cc)	3	P: type not reported S: antiemetics, gastric protection medications
Bozkaya et al. [8]	CT or MRI	CT or MRI	Bleomycin (15 mg in 5 mL saline, max. 30 mg) and lipiodol (10 mL, max. 20 mg)	4	P: type not reported S: sedation (pre), analgesia (pre and post) and antiemetics (post)
Cao et al. [41]	US, CT or MRI	CT	Bleomycin (16–32 mg)and lipiodol (10–15 mL) and gelatin sponge	NA	S: lidocaine 50 mg
Farges et al. [16]	US (153), CT (78), MRI (38), angiography (76) or diagnostic exploratory laparotomy (9)^a^	NA	NA	NA	NA
Firouznia et al. [42]	US, CT or MRI	US (17) or CT (3)	Polyvinyl alcohol particles (300–400 µm)	0	NA
Ji et al. [43]	NA	CT or MRI	Pingyangmycin (8 mg in 2 mL 5% glucose) and lipiodol (20 mL) and gelatin sponge particles (1–2 mm)	NA	S: sedation and analgesia
Kirnap et al. [53]	US (11), CT (6) or MRI	CT	Bleomycin (15 mg in 5 mL saline) and lipiodol (10 mL)	5	P: cephazolin 1g i.v. S: analgesics, sedatives
Li et al. [44]	US (75), CT (66), angiography (34), pathological diagnosis (21), ECT (20)^a^	US, CT or scintigraphy^a^	Gelfoam (100 mg) and lipiodol (8–16 mL)	T: 36^e^	NA
Li et al. [31]	CT or MRI	CT	Pingyangmycin (24 mg in 5 mL 1% lidocaine) and lipiodol (10 mL)	130	S: 2% lidocaine 5–10 mL, analgesia, antiemetics, reduced glutathione
Liu et al. [45]	CT or MRI	US, CT	Pingyangmycin and lipiodol	17^d^	S: local anaesthesia
Martin et al. [46]	US (4), CT (1) or scintigraphy (2)	US (3), CT (1), scintigraphy (1), angiography (1)^b^	50% isobutyl-2-cyanoacrylate and lipiodol (4 patients) or Gelatin sponge and thrombase (3 patients)	NA	NA
Ouyang et al. [47]	US, CT or angiography	NA	Bleomycin and lipiodol	NA	NA
Özden et al. [38]	MRI (25), biopsy (1)	CT or MRI	Bleomycin (15 mg in 5 mL non-ionic contrast agent) and lipiodol (10 mL)	8^c^	P: ampicillin, sulbactam and amoxicillin-clavulanate (pre and post) S: analgesics, antiemetics (pre and post)
Reading et al. [48]	US (24), angiography (22) or biopsy (12)^a^	Angiography	Gelfoam and sodium iothalamate and 50% dextrose	2^d^	P aminoglycosides and cephalosporins combined with metronidazole (pre and post) S: analgesia (post)
Srivastava et al. [49]	US, CT or MRI	US or CT	Polyvinyl alcohol particles and/or gelfoam and fibred steel coils	0	P: type not reported (post) S: analgesia (pre and post), sedation (pre), antiemetics (post)
Sun et al. [50]	US, CT or MRI	NA	Pingyangmycin (8–16 mg in 2 mL 5% glucose solution) and lipiodol	NA	S: rehydration
Wang et al. [51]	US, CT, angiography or pathological examinations	CT	Pingyangmycin (4–16 mg) and super liquefaction iodipin (5–20 mL), biological microspheres (300–500 µm, 1–5 mL)	NA	S: 1% lidocaine 2–5 mL
Zeng et al. [52]	US or CT	CT, chest photography	Pingyangmycin (8–24 mg in 2–10 mL lopamiro 300 and lipiodol	0	NA

Nr. TAE/TAL = number of patients needing more than one TAE/TAL session, T = total number of TAE/TAL procedures performed, P = prophylactic, S = supportive, ECT = emission computed tomography (e.g. PET or SPECT), NA = not available

^a^Reported for all patients with haemangioma, not only patients undergoing TAE/TAL

^b^Two patients lost to follow-up

^c^Includes one patient who underwent three TAE/TAL procedures

^d^Includes two patients who underwent three TAE/TAL procedures

^e^Including percutaneous embolization

### Success Rate

Multiple embolization/lipiodolization procedures were performed [8, 31, 38, 44, 45, 48, 53, 54] for different reasons in each study, but especially in patients with large tumours [8, 31, 38, 44, 53]. Failure of embolization/lipiodolization occurred in 4/1244 patients (0.3%) [48, 52]. Two of these patients were followed up without further treatment, one patient underwent exploratory laparotomy with open cannulation and embolization of the tumour [48], the last patient deviation from the protocol was deemed necessary and acceptable [52]. In four articles, the length of hospital stay was reported. Bozkaya et al. [8], Kirnap et al. [53], Özden et al. and Ji et al. reported 30 h, 28 h, 24–48 h and 2.2 days of hospital stay, respectively [38, 43]. This resulted in a weighted average hospital stay of 1.5 days.

### Tumour Size Analysis

Data from 1223/1284 (95.2%) patients in fifteen studies were reported following the RECIST guidelines; tumour size was reduced in 1100 patients (89.9%), stable in 89 patients (7.3%). Growth of tumour was reported in 34 patients (2.8%), all in one study [45]. The change in tumour size was measured after a weighted average of 8.6 months. In table S7, details on treatment of multiple haemangiomas are reported.

**Fig. 2:**
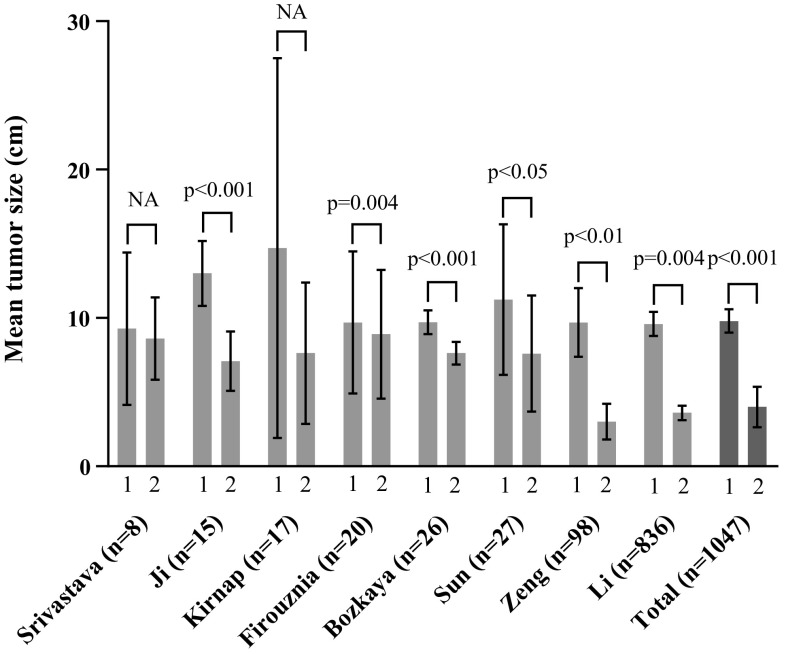
Tumour size pre- and post-TAE/TACE

The outcomes of the eight studies (including 1047 patients) that reported tumour size in cm before and after embolization/lipiodolization are shown in Fig. 2. The mean tumour diameter before TAE/TAL was 9.79 ± 0.79 cm; after TAE/TAL, this decreased to 4.00 ± 1.36 cm (*p* < 0.001). Volumetric analysis was performed in three papers. The article by Özden et al. demonstrated a reduction in median volume from 466 to 108 cm^3^; in the article by Bozkaya et al, the mean volume decreased from 446 to 244 cm^3^ [8, 38]. Kirnap et al. reported a mean volume reduction from 3716 to 746 cm^3^ [53].

### Symptoms and Symptom Relief

Of note, only one study used standardized tools to measure QoL or symptoms. Verbal rating scales were used by Kirnap et al. to measure the severity of pain [53], demonstrating that 9/23 patients became painless after TAE/TAL. The seven patients with initial severe symptoms had all improved. In most patients, 1192/1284 (92.8%), details on symptoms were reported. The majority, 1072/1192 (89.9%), was symptomatic at presentation. In 163/1152 patients (14.1%), a more detailed description of complaints was given.

Complaints were abdominal pain and discomfort in 65/163 patients (40.0%), bloating in 33/163 (20.2%), abdominal distension and mass effect in 4/163 (2.5%), multiple reasons in 56/163 (34.3%) and other in 5/163 (3.1%).

After treatment, symptoms were recorded for 1096/1284 (85.4%) patients. Complete resolution of symptoms occurred in 81/1096 patients (7.4%). In 999/1096 (91.1%), there was partial symptom relief, and in 16/1096 patients (1.5%), persistent or recurrent symptoms were reported. Five of the patients with persistent symptoms were eventually relieved of their symptoms, two by TAE/TAL and three by measures not reported [48]. Included articles generally did not state how long after TAE/TAL symptom relief occurred.

### Additional Procedures

After TAE/TAL, 35/1284 patients (2.7%) underwent surgery (Table 2) [45, 50]. Ji et al. used radiofrequency ablation (RFA) on month after transarterial embolization as part of the study protocol. As tumour size was measured after TAE/TAL but before RFA, this study was included in the analysis of tumour size [43].

### Complications

Complications occurred in 226/1284 (17.6%) patients. Postembolization syndrome was the main complication, occurring in 179/1284 patients (13.9%). Grade 3 complications were reported in 37/1284 patients (2.9%), mainly postprocedural pain [43], mild fever [50] and hepatic abscesses. Grade 3 complications and their management are reported in table S8. No mortality was reported.

## Discussion

TAE/TACE led to a mean reduction in size from 9.79 to 4.00 cm. Tumour size decreased in 89.9% of patients. Surgery was not required in 97.3% of patients. TAE/TAL appeared to be an effective method to diminish or resolve symptoms; in 999 patients (91.1%), TAE/TAL improved symptoms, and in 81 patients (7.4%), symptoms completely disappeared. Patients were followed for a weighted mean of 46.8 months. In 37 patients (2.9%), grade 3 complications occurred and no mortality was reported after TAE/TAL.

In current guidelines, TAE/TAL is not yet recommended as an alternative to surgery and is only considered to manage Kasabach–Merritt syndrome [25]. As this review shows promising results in regard to tumour size reduction and partial symptom relief, the role of TAE/TAL in the management of hepatic haemangioma might be reconsidered. The largest study on surgical treatment for hepatic haemangioma showed that surgery might provide symptomatic improvement of 44–87% of patients after a median follow-up of 2.8 years [55]. In comparison, in this review, partial or complete relief was present in 98.5% of patients after TAE/TAL after a median follow-up of 4 years. The occurrence of the three major morbidities, i.e. post-operative bleeding, bile leakage and liver failure was 1.8%, 5.1% and 1,6%, respectively. In contrast, the total number of grade 3 complications after TAE/TAL was 2.9%. After TAE/TAL, no mortality was reported compared to 0.2% mortality in the surgical study.

The largest study (836 patients) used glutathione as supportive treatment [31]. In humans, the only described indication of antioxidant use is in case of acetaminophen intoxication. There is no known indication for the use of glutathione in the setting of TAE/TAL. The complication rate of this prospective study was very low (2/836), which is interesting as most prospective studies tend to identify more complications than retrospective studies [56]. It is unclear whether the low rate of complications is associated with the administration of glutathione, the used embolization material (i.e. pingyangmycin and lipiodol) or perhaps the study size. A high volume of patients in an experienced centre is associated with better outcomes [57, 58].

Limitations of this review should be taken into account. Firstly, 35 articles, including a few large cohort studies, were excluded because these studies were not in English, French or German. Secondly, some of the included studies used parametric tests, while it was not clear whether the data were normally distributed, for example, the studies by Kirnap et al., *Srivastava* et al. and Sun et al. failed the so-called “95% range check” [49, 50, 59]. However, these data were taken into analysis as these were good quality studies based on the quality assessment. Finally, retrospective studies are often flawed by reporting bias. This might have led to an overestimation of the effects of TAE/TAL.

Future research should determine which treatment method is superior: transarterial (chemo-)embolization or lipiodolization. No further predictive factors of treatment success could be identified, due to the heterogeneity of studies. Ideally, TAE/TAL should be compared to conservative management and surgery. Moreover, QoL deserves a prominent role in future research because almost all patients undergoing treatment have symptoms. QoL might be evaluated by, for example, the EORTC QLQ-C30 questionnaire.

## Conclusion

When treatment is indicated for liver haemangiomas (e.g. large tumour size and/or symptoms), TAE/TAL appears to be effective to decrease tumour size. It may provide relief of symptoms, however, no large prospective studies using QoL questionnaires are available. In this review, TAE/TAL was considered a safe treatment method of liver haemangiomas TAE/TAL potentially serves as a viable alternative to resection.

## Electronic supplementary material

Below is the link to the electronic supplementary material.

Supplementary material 1 (DOCX 65 kb)
