# Supplementary material for: Transarterial (Chemo-)Embolization and Lipiodolization for Hepatic Haemangioma
Source: Cardiovasc Intervent Radiol. 2019 Feb 19;42(6):800–11. doi: 10.1007/s00270-019-02169-x (PMC6503075; doi:10.1007/s00270-019-02169-x)
Supplement: Supplementary file 1 — Supplementary material 1 (DOCX 65 kb) [file 270_2019_2169_MOESM1_ESM.docx]

**Supplementary file 1**

**Disclosure**

The International Society for the Study of Vascular Anomalies (ISSVA) suggests the use of the term “venous malformation” instead of adult hepatic haemangioma.^1^ However, in clinical practice, the term haemangioma is still used to described these lesions. We do agree that terminology should be applied according to current guidelines. However, to increase the understandability of our paper we have used the term “hepatic haemangioma”.

**Standardised in- and exclusion forms**

Pubmed (MEDLINE)

("Hemangioma"[Mesh] OR hemangioma*[tiab] OR haemangioma*[tiab])  AND ("Embolization, Therapeutic"[Mesh:NoExp] OR "Chemoembolization, Therapeutic"[Mesh] OR transarterial embolization*[tiab] OR transarterial chemoembolization*[tiab]  OR transarterial embolization*[tiab] OR transarterial chemoembolization*[tiab] OR TAE[tiab] OR TACE[tiab] OR chemoembolization*[tiab] OR embolization*[tiab] OR chemoembolisation*[tiab] OR embolisation*[tiab]) AND ("Liver"[Mesh] OR "Liver Neoplasms"[Majr:NoExp] OR liver[tiab] OR hepatic[tiab])

Embase (OVID):

| # | Searches |
| --- | --- |
| 1 | hemangioma/ or liver hemangioma/ or (hemangioma* or haemangioma*).ti,ab,kw. |
| 2 | artificial embolization/ or chemoembolization/ or (transarterial embolization* or transarterial chemoembolization* or transarterial embolization* or transarterial chemoembolization* or TAE or TACE or chemoembolization* or embolization* or chemoembolisation* or embolisation*).ti,ab,kw. |
| 3 | exp liver/ or exp liver tumor/ or (liver or hepatic).ti,ab,kw. |
| 4 | 1 and 2 and 3 |

**Exclusion criteria**

- Infantile hepatic haemangioma (children under the age of 18)
- Ruptured haemangioma
- Extra-hepatic haemangioma
- Patients with concomitant malignancies
- Non-TAE or –TAL techniques
  - Hepatic arterial ligation
  - Portal vein embolization
  - Corticosteroids
  - Interferon alpha2a
  - Radiotherapy
  - Radiofrequency ablation
  - Liver transplantation
- Inherited disease
- Syndromal disease
- Severe systemic disease
- Reviews

**Inclusion criteria**

- Patients with hepatic haemangioma above 18 years of age that have undergone TAE/TAL

**Baseline characteristics**

- Author and institution
- Publication date
- Study design
- Age
- Gender
- Reason for TAE/TAL
- TAE/TAL details (material, number of procedures needed)
- Total number of patients
- Number of patients with haemangioma
- Number of patients undergoing TAE/TAL for haemangioma
- Number of tumours per patient (solitary of multiple)
- Comorbidity
- Liver lobe
- Pre-operative (any TAE/TAL carried out followed by (planned) surgery within one month) or elective (TAE/TAL procedures which were not performed in order to reduce intra-operative blood loss or stop bleeding in the emergency setting, but as a planned therapeutic treatment for haemangioma) procedure

**Outcome measures**

- Symptoms at presentation (abdominal pain and discomfort, bloating and abdominal distension, mass effect)
- Follow-up duration (months)
- Tumour size before procedure (in cm)
- Tumour size after procedure (in cm)
- Imaging before and after procedure (i.e. US, CT, MRI, liver scintigraphy such as hepatic blood pool scanning, angiography without intervention)
- Technical success was defined as a haemangioma treated according to protocol, were the haemangioma was addressed completely (embolic material or lipiodol and chemotherapeutic agent were deposited in the haemangioma), sometimes verified by the identification of lipiodol of embolic material in the tumour^2^

Technical failure was defined as the case in which technical success could not be achieved

- Response evaluation criteria in solid tumors (RECIST) criteria^3^
- Reduction of VAS-score (visual analogue score)
- Improvement on QoL questionnaires (every type of validated questionnaire)
- Decreased or improved symptoms

Complications following the Cardiovascular and Interventional Radiological Society of Europe (CIRSE) classification^4^

Complications were classified as follows:

1 Complication during the procedure which could be solved within the same session; no additional therapy, no postprocedure sequelae, no deviation from the normal posttherapeutic course

2 Prolonged observation including overnight stay (as a deviation from the normal post-therapeutic course >48 h); no additional postprocedure therapy, no postprocedure sequelae

3 Additional postprocedure therapy or prolonged hospital stay (>48 h) required; no postprocedure sequelae

4 Complication causing a permanent mild sequelae (resuming work and independent living)

5 Complication causing a permanent severe sequelae (requiring ongoing assistance in daily life)

6 Death

- Type of complication
  - Hepatic abscess
  - Liver dysfunction
  - Necrosis of the normal liver
  - Sepsis
  - Biliary tree damage
  - Renal failure
  - Bowel infarction
  - Post-embolization syndrome (a combination of abdominal pain and distension, fever and nausea)
- Need for further surgery (yes/no)
- Reason to perform elective TAE/TAL
- Reason to perform surgery
- Reason not to perform surgery
- Number of TAE/TAL procedures needed per patient
- Hospital stay duration
- Prophylactic and supportive care
- TAE-/TAL-related mortality
- Overall mortality

# Supplementary file 2

**Table S1: Quality assessment**

| **Downs and Black** | **Reporting** | | | | | | | | | | **External validity** | | | **Internal validity / bias** | | | | | | | **Internal validity / confounding** | | | | | | **Power** | **Total** |
| --- | --- | --- | --- | --- | --- | --- | --- | --- | --- | --- | --- | --- | --- | --- | --- | --- | --- | --- | --- | --- | --- | --- | --- | --- | --- | --- | --- | --- |
| Akhlaghpoor *et al.* (2018) | 1 | 1 | 1 | 1 | 1 | 1 | 1 | 0 | 0 | 1 | 0 | 0 | 0 | 0 | 0 | 0 | 0 | 1 | 1 | 1 | 0 | 0 | 0 | 0 | 0 | 0 | 0 | **11** |
| Bozkaya *et al*. (2014)^5^ | 1 | 1 | 0 | 1 | 1 | 1 | 1 | 1 | 0 | 1 | 0 | 0 | 0 | 0 | 0 | 1 | 0 | 1 | 1 | 0 | 0 | 0 | 0 | 0 | 0 | 0 | 0 | **11** |
| Cao *et al.* (2000)^6^ | 0 | 0 | 0 | 1 | 1 | 0 | 0 | 0 | 0 | 0 | 0 | 0 | 0 | 0 | 0 | 1 | 1 | 1 | 1 | 0 | 0 | 0 | 0 | 0 | 0 | 0 | 0 | **6** |
| Farges *et al.* (1995)^7^ | 1 | 0 | 1 | 0 | 1 | 1 | 0 | 0 | 0 | 0 | 0 | 0 | 0 | 0 | 0 | 1 | 0 | 1 | 1 | 1 | 1 | 1 | 0 | 0 | 0 | 0 | 0 | **10** |
| Firouznia *et al.* (2014)^8^ | 1 | 1 | 1 | 1 | 1 | 1 | 1 | 1 | 0 | 1 | 0 | 0 | 0 | 0 | 0 | 1 | 0 | 0 | 1 | 0 | 0 | 0 | 0 | 0 | 0 | 0 | 0 | **11** |
| Ji *et al.* (2016)^9^ | 1 | 1 | 1 | 1 | 1 | 1 | 1 | 1 | 0 | 1 | 0 | 0 | 1 | 0 | 0 | 1 | 1 | 1 | 1 | 0 | 0 | 0 | 0 | 0 | 0 | 0 | 0 | **14** |
| Kirnap *et al.* (2018) | 1 | 0 | 0 | 1 | 1 | 1 | 1 | 1 | 0 | 1 | 0 | 0 | 0 | 0 | 0 | 1 | 1 | 1 | 1 | 0 | 0 | 0 | 0 | 0 | 0 | 0 | 0 | **11** |
| Li *et al.* (1997)^10^ | 1 | 0 | 0 | 0 | 0 | 0 | 0 | 0 | 0 | 0 | 0 | 0 | 0 | 0 | 0 | 1 | 0 | 1 | 1 | 0 | 0 | 0 | 0 | 0 | 0 | 0 | 0 | **4** |
| Li *et al.* (2015)^11^ | 1 | 1 | 1 | 1 | 1 | 1 | 1 | 1 | 0 | 1 | 0 | 0 | 0 | 0 | 0 | 1 | 0 | 1 | 1 | 1 | 0 | 0 | 0 | 0 | 0 | 0 | 0 | **13** |
| Liu *et al.* (2017)^12^ | 1 | 1 | 1 | 1 | 1 | 1 | 0 | 0 | 0 | 1 | 0 | 0 | 1 | 0 | 0 | 1 | 0 | 1 | 1 | 0 | 0 | 0 | 0 | 0 | 0 | 0 | 0 | **11** |
| Martin *et al.* (1986)^13^ | 0 | 0 | 0 | 0 | 1 | 1 | 0 | 0 | 1 | 0 | 0 | 0 | 0 | 0 | 0 | 1 | 0 | 1 | 1 | 0 | 0 | 0 | 0 | 0 | 0 | 0 | 0 | **6** |
| Ouyang *et al.* (2001)^14^ | 1 | 1 | 1 | 1 | 1 | 1 | 0 | 0 | 0 | 0 | 0 | 0 | 1 | 0 | 0 | 1 | 1 | 1 | 1 | 0 | 0 | 0 | 0 | 0 | 0 | 0 | 0 | **11** |
| Özden *et al.* (2017)^15^ | 1 | 1 | 0 | 1 | 1 | 1 | 0 | 1 | 0 | 0 | 0 | 0 | 0 | 0 | 0 | 0 | 0 | 1 | 1 | 0 | 0 | 0 | 0 | 0 | 0 | 0 | 0 | **8** |
| Reading *et al.* (1988)^16^ | 1 | 0 | 0 | 1 | 1 | 1 | 0 | 0 | 0 | 0 | 0 | 0 | 0 | 0 | 0 | 0 | 0 | 1 | 1 | 0 | 1 | 1 | 0 | 0 | 0 | 0 | 0 | **8** |
| Srivastava *et al.* (2001)^17^ | 0 | 1 | 1 | 1 | 1 | 1 | 1 | 0 | 0 | 0 | 0 | 0 | 0 | 0 | 0 | 1 | 1 | 1 | 1 | 0 | 0 | 0 | 0 | 0 | 0 | 0 | 0 | **10** |
| Sun *et al.* (2015)^18^ | 1 | 1 | 1 | 1 | 2 | 1 | 1 | 1 | 1 | 0 | 0 | 0 | 0 | 0 | 0 | 1 | 1 | 0 | 1 | 1 | 0 | 0 | 0 | 0 | 0 | 1 | 0 | **15** |
| Wang *et al.* (2016)^19^ | 1 | 0 | 1 | 1 | 1 | 1 | 0 | 0 | 0 | 0 | 0 | 0 | 0 | 0 | 0 | 1 | 0 | 0 | 1 | 0 | 0 | 0 | 0 | 0 | 0 | 0 | 0 | **7** |
| Zeng *et al.* (2004)^20^ | 1 | 1 | 1 | 1 | 1 | 1 | 1 | 1 | 0 | 0 | 0 | 0 | 0 | 0 | 0 | 1 | 1 | 1 | 1 | 0 | 0 | 0 | 0 | 0 | 0 | 0 | 0 | **12** |
| Downs and Black is a checklist for assessment of methodological quality of randomised and non-randomised studies, consisting of a total of 27 questions. The maximum score is 31, all items are scored 0 or 1 except the fifth item on reporting (is scored 0, 1 or 2) and the last item on power (is scored 0-5).^21^ | | | | | | | | | | | | | | | | | | | | | | | | | | | | |

**Table S2 Reported comorbidity in cohort studies**

| **Author** | **Reported comorbidity** |
| --- | --- |
| Akhlaghpoor *et al.* (2018) | NA |
| Bozkaya *et al.* (2014)^5^ | NA |
| Cao *et al.* (2000)^6^ | NA |
| Farges *et al.* (1995)^7^ | **Comorbidity:** Biliary lithiasis (18), gastroduodenal ulcer / gastritis (8), benign liver tumour (5 cyst, 2 adenoma), hiatal hernia (6), colopathy (6), hepatitis/cirrhosis (5), diverticular disease (4), gallbladder polyps (2), pancreas divisum (1), pericarditis (1), Fitz-Hugh-Curtis syndrome (1), pregnancy (1), vertebral angioma (1), premenstrual syndrome (1), renal cysts (1), chronic pelvic suppuration (1), endometriosis (1), renal cyst (1)^a^  **Malignancy in medical history:** breast cancer (8), kidney cancer (5), colon cancer (4), stomach cancer (2).  **Haemangioma-related complications:** Kasabach-Merritt syndrome with spontaneous haemorrhage (2), Budd-Chiari syndrome with tense ascites |
| Firouznia *et al.* (2014)^8^ | NA |
| Ji *et al.* (2016)^9^ | Liver cyst (3), chronic hepatitis B (2), diabetes (1) |
| Kirnap *et al.* (2018) | NA |
| Li *et al.* (1997)^10^ | 2 HBsAg +, 1 HBsAb + |
| Li *et al.* (2015)^11^ | None |
| Liu *et al* (2017)^12^ | NA |
| Martin *et al.* (1986)^13^ | None |
| Ouyang *et al.* (2001)^14^ | None |
| Özden *et al.* (2017)^15^ | Cystadenoma (1), peptic ulcer (2), cholecystolithiasis (2) |
| Reading *et al.* (1988)^16^ | None |
| Srivastava *et al.* (2001)^17^ | NA |
| Sun *et al.* (2015)^18^ | Cholecystitis (3, 2 with gall bladder stones), associated liver cyst (6), hypertension (2), diabetes (1), gallbladder polyps (1), uterine fibroids (1) |
| Wang *et al.* (2016)^19^ | NA |
| Zeng *et al.* (2004)^20^ | NA |
| NA = not available  a Reported for all 163 patients, nu only patients undergoing TAE/TAL | |

**Table S3 Baseline characteristics of patients included in case reports**

|  | **T/H/E** | **S/M** | **L/R/B** | **Age** | **F/M** | **Follow-up** | **KMS** |
| --- | --- | --- | --- | --- | --- | --- | --- |
| Akamatsu *et al.* (2010)^22^ | 1/1/1 | 1/0 | 0/1/0 | 38.0 | 0/1 | 27 | Yes, 1 |
| Althaus *et al.* (1996)^23^ | 1/1/1 | 0/1 | 0/0/1 | 29.0 | 1/0 | 61 | No |
| Bozkaya *et al.* (2015)^24^ | 1/1/1 | 1/0 | 0/0/1 | 34.0 | 1/0 | 7 | Yes, 1 |
| Buffet *et al.* (1982)^25^ | 3/3/1 | 0/1 | 0/0/1 | 42.0 | 1/0 | 42 | No |
| Deutsch *et al.* (2001)^26^ | 3/3/3 | 3/0 | 2/1/0 | 57.0 | 2/1 | 19, NA: 2 | No |
| Fung *et al.* (2013)^27^ | 1/1/1 | 1/0 | 1/0/0 | 49.0 | 1/0 | 19 | Yes, 1 |
| Giavroglou *et al.* (2003)^28^ | 4/4/4 | 2/2 | 1/2/0^a^ | 51.3 | 3/1 | 23.8 | No |
| Igarashi *et al.* (2018)^29^ | 1/1/1 | 1/0 | 1/0/0 | 73.0 | 1/0 | 24 | No |
| Jin *et al.* (2014)^30^ | 1/1/1 | 1/0 | 0/1/0 | 44.0 | 1/0 | 7 | No |
| Kapoor *et al.* (2005)^31^ | 1/1/1 | 1/0 | 0/1/0 | 50.0 | 1/0 | 42 | No |
| Koniaris *et al.* (2003)^32^ | 1/1/1 | 1/0 | 0/0/1^c^ | 35.0 | 1/0 | 68 | No |
| Malagari *et al.* (2009)^33^ | 2/2/2 | 2/0 | NA | 63.0 | 2/0 | 24 | Yes, 2 |
| Meguro *et al.* (2008)^34^ | 1/1/1 | 0/1 | 0/0/1 | 45.0 | 1/0 | 22 | Yes, 1 |
| Mohan *et al.* (2007)^35^ | 1/1/1 | 1/0 | 0/1/0 | 36.0 | 0/1 | NA | No |
| Moschouris *et al.* (2010)^36^ | 10/1/1 | 0/1 | NA | 35.0 | 1/0 | 1.5 | No |
| Nobuoka *et al.* (2004)^37^ | 1/1/1 | 1/0 | 0/0/1 | 51.0 | 1/0 | 42 | No |
| Pachera *et al.* (2009)^38^ | 1/1/1 | 0/1 | 0/0/1 | 45.0 | 1/0 | 62 | Yes, 1 |
| Roche *et al.* (1978)^39^ | 35/2/2 | 2/0 | 0/1/0, NA: 1 | NA | NA | 11 | No |
| Szejnfeld *et al.* (2015)^40^ | 3/3/3 | NA | NA | 57.3 | 2/1 | 2 | No |
| Tanaka *et al.* (2002)^41^ | 2/2/1 | 0/1 | 0/0/1^d^ | 32.0 | 1/0 | 124 | No |
| Tarazov *et al.* (1990)^42^ | 1/1/1 | NA | NA | 38.0 | 1/0 | 39 | No |
| Tarazov *et al.* (1993)^43^ | 2/2/2 | 0/2 | 1/0/1 | 38.0 | 2/0 | 26 | No |
| Xu *et al.* (2010)^44^ | 11/11/1 | 0/1^e^ | 0/1/0 | 53.0 | 1/0 | 70 | No |
| Yang *et al.* (2017)^45^ | 1/1/1 | 1/0 | NA | 33.0 | 0/1 | 58 | Yes, 1 |
| Zhou *et al.* (2013)^46^ | 1/1/1 | 1/0 | 1/0/0 | 21.0 | 0/1 | 76 | No |
| **Total** | **90/48/35** | **20/11** | **7/10/9** | **46.5^b^** | **26/7** | **32.3^b^** | **8 patients with KMS** |
| T/H/E = total number of patients / patients with haemangioma / patients with haemangioma treated with TAE/TAL, Age = mean age in years, F/M = female / male, L/R/B = left / right / bilateral, Follow-up = mean follow-up in months, KMS = Kasabach-Merritt  a Unclear method of reporting: one patient with the largest tumour in the left lobe, but unclear where the other tumours are located  b Weighted average  c Unclear method of reporting: right and left lobe are replaced by haemangioma  d Haemangiomatosis of the entire liver  e At the time TAE/TAL was performed, only haemangioma of the right liver lobe was known | | | | | | | |

**Table S4 Diagnostic and treatment methods used in case reports**

|  | **Diagnostic methods before TAE/TAL** | **Diagnostic methods after TAE/TAL** | **TAE/TAL material** | **Nr. TAE/ TAL** | **Farmacotherapeutics** |
| --- | --- | --- | --- | --- | --- |
| Akamatsu *et al.* (2010)^22^ | CT | CT | Coils and gelfoam | 1 | NA |
| Althaus *et al.* (1996)^23^ | US, MRI | CT, MRI | Polyvinyl alcohol (150-250 µm) | 1 | S: nitroglycerin 100-200 µg, percutaneous celiac plexus nerve block, morphine |
| Bozkaya *et al.* (2015)^24^ | CT, bone marrow aspiration biopsy | CT | Bleomycin (15 mg) and lipiodol (20 mL) | 1^a^ | NA |
| Buffet *et al.* (1982)^25^ | Scintigraphy, laparoscopy, | Angiography, scintigraphy | NA | 0 | NA |
| Deutsch *et al.* (2001)^26^ | CT (3) | NA | Polyvinyl alcohol (1x), particle embolization (1x), NA: 1 | 0 | NA |
| Fung *et al.* (2013)^27^ | US, CT | CT | Polyvinyl alcohol (350 µm, 1x), gelfoam and coils and Embosphere (500 µm, 1x)^b^ | 1 | NA |
| Giavroglou *et al.* (2003)^28^ | US (3), CT (4), MRI (4), technetium-label red blood cell study (1), exploratory laparotomy and open biopsy (1) | US | Polyvinyl alcohol (150-250 µm) | 0 | P: antibiotics (post, 1x), tazocin® (Piperacillin and tazobactam) 4.5 mg four times daily (post, 1x) |
| Igarashi *et al.* (2018)^29^ | CT, PET, esophago-gastroduodenoscopy | CT | Gelfoam particles | 1 | NA |
| Jin *et al.* (2014)^30^ | NA | CT, MRCP and ERCP | Bleomycin-iodinated oil | 0 | NA |
| Kapoor *et al.* (2005)^31^ | US, CT | CT | Steel coils | 0 | NA |
| Koniaris *et al.* (2003)^32^ | CT | Biliary DISIDA scan, ERCP | Absolute ethanol (30 mL) and polyvinyl alcohol (700 µm) and Gianturco embolization coils | 0 | NA |
| Malagari *et al.* (2009)^33^ | CT, US, MRI (2) | US, CT | Embospheres (Biosphere medical, Rockland Mass., USA)® (40-300 µm, 2x) and coil (1x) | 0 | S: correction of coagulation parameters (pre) |
| Meguro *et al.* (2008)^34^ | CT, MRI | CT | NA | 1 | NA |
| Mohan *et al.* (2007)^35^ | US, CT | NA | Polyvinyl alcohol (300-500 µm) | 0 | P: antibiotics (pre), intravenous ciprofloxacin and metronidazole (post)  S: conscious sedation, oral paracetamol (post), diclofenac (post), sodium (post) |
| Moschouris *et al.* (2010)^36^ | US, MRI | US, MRI | Embozene microspheres®(Embozene; CeloNova BioSciences, Newnan, GA) (40, 100 and 400 µm) | 0 | NA |
| Nobuoka *et al.* (2004)^37^ | US, CT | US, CT | Gelatin sponge | 0 | NA |
| Pachera *et al.* (2009)^38^ | NA | CT, plasma disappearance rate of indocyanin green | NA | 1 | NA |
| Roche *et al.* (1978)^39^ | Scintigraphy | Scintigraphy and angiography | Gelatin sponge fragments with thrombase and epsilon-amino-caproïque | 0 | S: droperidol and phenoperidine |
| Szejnfeld *et al.* (2015)^40^ | CT and/or MRI | CT, MRI | Absolute ethanol (8 mL) and lipiodol (2 mL) | NA | S: conscious sedation, 0,5% lidocaine 2 mL |
| Tanaka *et al.* (2002)^41^ | Chest radiograph, doppler-US (cardiac output), ECG | Cardiothoracic ratio (CTR) | Steel coils (40 coils of 2x12 mm, 40 coils of 3x10 mm, 2 coils of 3x40 mm) | NA | NA |
| Tarazov *et al.* (1990)^42^ | US, scintigraphy, needle biopsy, endoscopy | Angiography, endoscopy | Spongostan®(Ferrosan Co., Copenhagen, Denmark) (1 cm^3^ of 2x2x5 mm particles) and three modified Gianturco coils ®(Cook Co., Bloomington, IN) (stainless steel guidewire 0.95 mm in diameter, 5-15 cm long when stretched and 5-10 spires of 0.3-0.8 cm) | 1 | NA |
| Tarazov *et al.* (1993)^43^ | US (2), CT (1), angiography (1) | US, CT, angiography, US-guided biopsy | Gelatin sponge (2 mL, 2x2 mm^2^) and a steel coil (1x), gelatin sponge (1x), 2 steel coils (1x) | 1 | NA |
| Xu *et al.* (2010)^44^ | NA | NA | NA | 1 | NA |
| Yang *et al.* (2017)^45^ | US, CT | CT-angiography | NA | 1 | NA |
| Zhou *et al.* (2013)^46^ | US, CT, MRI | CT | Lipiodol | 0 | NA |
| Nr. TAE/TAL = number of patients needing more than one TAE/TAL session, P = prophylactic, S = supportive  a Three TAE/TAL sessions performed  b Two TAE/TALs in the same patient with different materials | | | | | |

**Table S5 Reason for treatment and outcomes in case reports**

|  | Indications for TAE/TAL | Reasons (not) to perform surgery | Tumour size reduction | Follow-up | Symptoms before TAE/TAL | S.I. | Complications |
| --- | --- | --- | --- | --- | --- | --- | --- |
| Akamatsu *et al.* (2010)^22^ | NA | Pre-operative (1): possible due to size reduction | 16880 mL - 8260 mL | 2 | M: 1 | Improved QoL^f^ | None |
| Althaus *et al.* (1996)^23^ | M: 1 | Elective: reason not stated | R: 1, Ø 9 – 6 | 16 | PD: 1 | I: 1 | Grade 1 (1): mild PES |
| Bozkaya *et al.* (2015)^24^ | NA | Elective: unresectable tumour | R: 1, 45% reduction | 6 | PD: 1 | D: 1 | Grade 3 (1): PES with symptomatic treatment |
| Buffet *et al.* (1982)^25^ | NA | Elective: unresectable tumours | S: 1 | 6 | ME: 1 | NA | Grade 3 (1): fever for 4 months with antibiotic treatment, pain at thorax basis |
| Deutsch *et al.* (2001)^26^ | NA | Elective: reason not stated | R: 1, Ø 19 – 12, NA: 2 | NA | PD: 1 ME: 1 M: 1 | D: 2, P: 1 | Grade 3 (1): transfusion due to low Hb  Grade 1(1): femoral hematoma and transient abdominal and back pain, none (1) |
| Fung *et al.* (2013)^27^ | AS: 1 | Elective: reason not stated | S: 1^a^ | 1 | M: 1 | D: 1 | Grade 1 (1): mild abdominal pain |
| Giavroglou *et al.* (2003)^28^ | AS: 4 | Elective: reason not stated | S: 2, Ø 9.5 – 8.0, NA: 2^c^ | 45 | PD: 3 | D: 3, I: 1 | Grade 3 (1): mild local pain treated by antibiotics iv., None (3) |
| Igarashi *et al.* (2018)^29^ | NA | Elective: directed at arterio-portal shunt, to reduce portal hypertension | NA | NA | M: 1 | D: 1 | NA |
| Jin *et al.* (2014)^30^ | NA | Pre-operative (1): suspected malignancy (sclerosing cholangitis) | R: 1, Ø 9 – 6^d^ | 72 | NA | D: 1 | Grade 3 (1): sclerosing cholangitis |
| Kapoor *et al.* (2005)^31^ | NA | Pre-operative (1): abdominal compartment syndrome and enlarging haemangioma | G: 1 | 2 | M: 1 | P: 1 | NA |
| Koniaris *et al.* (2003)^32^ | NA | Pre-operative (1): reason not stated | NA | NA | PD: 1 | NA | Grade 3 (1): bile duct fibrosis, acute pain and decreased haematocrit |
| Malagari *et al.* (2009)^33^ | AS: 1 | Elective: reason not stated | S: 2 | 24 | M: 1 | NA | Too proximal release of coils Technical failure, not complication |
| Meguro *et al.* (2008)^34^ | NA | Pre-operative (1): TAE/TAL not effective | S: 1 | NA | PD: 1 | P: 1 | NA |
| Mohan *et al.* (2007)^35^ | NA | Elective: reason not stated | NA | NA | PD: 1 | NA | Grade 3 (1): mild pain and fever treated with paracetamol and diclofenac |
| Moschouris *et al.* (2010)^36^ | NA | Elective: reason not stated | NA | NA | NA | NA | NA |
| Nobuoka *et al.* (2004)^37^ | NA | Pre-operative (1): intratumoural haemorrhage | NA | NA | PD: 1 | D: 1 | Grade 1 (1): abdominal pain and mild fever |
| Pachera *et al.* (2009)^38^ | M: 1 | Pre-operative (1): persistent symptoms | S: 1 | NA | SO: 1 | P: 1 | NA |
| Roche *et al.* (1978)^39^ | L: 1 | Elective: reason not stated | S: 2 | 3,5 | O: 1 | NA | Grade 1 (30): fever, Grade 1 (12): severe pain^g^ |
| Szejnfeld *et al.* (2015)^40^ | H: 3 | Elective: unresectable tumour | S:3, Ø 17.3 – 14.2 | 2-3 | SO: 3 | Improved QoL | Grade 3 (3): moderate pain treated with fentanyl, ondansetron and cefazolin |
| Tanaka *et al.* (2002)^41^ | NA | Elective: reason not stated | NA | NA | M: 1 | D: 1 | Grade 1 (1): refractory nausea and vomiting |
| Tarazov *et al.* (1990)^42^ | NA | Pre-operative (1): splenectomy, unresectable hepatic tumours | Only decrease in liver size reported | NA | M: 1 | D:1 | Grade 1 (1): post-embolization syndrome Grade 6 (F): due to Ewing sarcoma, not a consequence of TAE/TAL |
| Tarazov *et al.* (1993)^43^ | NA | Elective: refusal to surgery (1), unresectable tumour (1) | R: 2 | 26 | M: 1 O: 1 | NA | Grade 2 (1): acute ischemic cholecystitis treated conservatively, None (1) |
| Xu *et al.* (2010)^44^ | NA | Pre-operative (1): persistent symptoms and stable tumour size | S: 1 | NA | NA | P: 1 | Grade 6 (1): after resection, not a consequence of TAE/TAL |
| Yang *et al.* (2017)^45^ | L: 1 | Pre-operative (1): symptoms | S: 1 | NA | A: 1 | NA | NA |
| Zhou *et al.* (2013)^46^ | NA | Pre-operative (1): intestinal obstruction | S:1, Ø 31.5 – 26.5)^b^ | 1 | PD: 1 | NA | NA |
| **Total** | **AS: 6 L: 2 H: 3 M: 2** | **12 patients pre-operative** | **R: 6, S: 16, G: 1** | **16,3^e^** | **PD: 11**  **M: 9 O: 2 ME: 2**  **A: 1 SO: 1** | **D: 10, I: 2, P: 5** | **Grade 6: 2 Grade 3: 10**  **Grade 2: 1**  **Grade 1: 6** |
| Tumour size = (mean) tumour size before TAE/TAL – after TAE/TAL in cm, Timing = (mean) timing of follow-up imaging for tumour size after last TAE/TACE session in months, Surgery y/n = number of patients treated with surgery after TAE/TAL / number of patients treated with TAE/TAL alone , AS = in order to alleviate symptoms, E = enlarging tumour L= large tumour, H = haemorrhage, high bleeding risk, O = other , R = reduction (>30%) decrease of tumour size , S = stable (<30%) decrease and (<20%) increase of tumour size, G = growth (>20%) increase of tumour size, PD = pain or discomfort, B = bloating or abdominal distension, M = multiple, ME = mass effect including dyspepsia and palpable masses, SO = symptoms not otherwise specified, A = asymptomatic, D = disappearance of symptoms, I = improvement of symptoms, P = persistent or recurrent symptoms, QoL = quality of life a Only reported after first TAE/TAL session, not after second session  b Tumour size after TAE/TAL measured at resection  c Two patients with no tumour size after TAE/TAL reported, one and four months follow-up. Tumour sizes before TAE/TAL were 10,5 and 23 cm d Tumour size after TAE/TAL measured at exploratory laparotomy  e Weighted average  f Reported after surgery  g Only reported for all patients undergoing TAE/TAL, not only patients with haemangioma. Excluded from analysis. | | | | | | | |

**Table S6 Comorbidity and hospital stay reported in case reports**

|  | **Comorbidity** | **Hospital stay** |
| --- | --- | --- |
| Akamatsu *et al.* (2010)^22^ | None | NA |
| Althaus *et al.* (1996)^23^ | None | NA |
| Bozkaya *et al.* (2015)^24^ | NA | NA |
| Buffet *et al.* (1982)^25^ | None | NA |
| Deutsch *et al.* (2001)^26^ | Prostate cancer (1), anaemia and uterine fibroids (1), hypertension, gastro-oesophageal reflux, bilateral tubal ligation and chronic anaemia (1) | 2 days |
| Fung *et al.* (2013)^27^ | None | NA |
| Giavroglou *et al.* (2003)^28^ | Echinococcal cyst 20 years prior to presentation (1) | 2 days and 3 days |
| Igarashi *et al.* (2018)^29^ | Ascites and portal hypertension, related to haemangioma (1) | 8 days |
| Jin *et al.* (2014)^30^ | NA | NA |
| Kapoor *et al.* (2005)^31^ | NA | NA |
| Koniaris *et al.* (2003)^32^ | NA | NA |
| Malagari *et al.* (2009)^33^ | Chronic hepatitis C (1) | NA |
| Meguro *et al.* (2008)^34^ | NA | NA |
| Mohan *et al.* (2007)^35^ | NA | 7 days |
| Moschouris *et al.* (2010)^36^ | NA | NA |
| Nobuoka *et al.* (2004)^37^ | NA | NA |
| Pachera *et al.* (2009)^38^ | None | NA |
| Roche *et al.* (1978)^39^ | Haemophilia A (1) | NA |
| Szejnfeld *et al.* (2015)^40^ | NA | Less than 12 hours |
| Tanaka *et al.* (2002)^41^ | Atrial fibrillation and heart failure (1) | NA |
| Tarazov *et al.* (1990)^42^ | NA | 14 days |
| Tarazov *et al.* (1993)^43^ | NA | NA |
| Xu *et al.* (2010)^44^ | NA | NA |
| Yang *et al.* (2017)^45^ | NA | NA |
| Zhou *et al.* (2013)^46^ | NA | NA |

**Table S7 Treatment of multiple haemangioma in cohort studies**

| **Author** | **Methods of treatment and follow-up of multiple haemangioma** |
| --- | --- |
| Akhlaghpoor *et al.* (2018) | Multiple haemangiomas underwent multiple TAE/TAL procedures, data reported per haemangioma (not per patient) |
| Bozkaya *et al.* (2014)^5^ | Multiple TAE/TAL procedures in four patients with diffuse haemangioma, all lesions included in tumour size analysis |
| Cao *et al.* (2000)^6^ | NA |
| Farges *et al.* (1995)^7^ | Unclear if patients with multiple haemangioma underwent multiple TAE/TAL procedures |
| Firouznia *et al.* (2014)^8^ | Only one tumour per patient embolized, so in five patients with multiple haemangioma only one is embolized. Unclear if only embolized haemangioma are included in tumour size analysis |
| Ji *et al.* (2016)^9^ | Only one tumour per patient embolized and included in tumour size analysis |
| Kirnap *et al.* (2018) | Three patients underwent simultaneous treatment of bilateral haemangioma. In patients with multiple haemangioma, non-intervened haemangioma were also analysed, but separately |
| Li *et al.* (1997)^10^ | NA |
| Li *et al.* (2015)^11^ | Multiple TAE/TAL procedures performed in patients with multiple haemangioma. Unclear if all embolized lesions are included in tumour size analysis |
| Liu *et al.* (2017)^12^ | NA |
| Martin *et al.* (1986)^13^ | Only changes in liver size reported for patients undergoing multiple TAE/TAL procedures |
| Ouyang *et al.* (2001)^14^ | NA |
| Özden *et al.* (2017)^15^ | Only largest tumour embolized if multiple haemangioma were present. Unclear if only embolized haemangioma are included in tumour size analysis. |
| Reading *et al.* (1988)^16^ | NA |
| Srivastava *et al.* (2001)^17^ | NA |
| Sun *et al.* (2015)^18^ | NA |
| Wang *et al.* (2016)^19^ | NA |
| Zeng *et al.* (2004)^20^ | NA |

**Table S8 Management of grade 3 complications in cohort studies**

| **Grade 3 complication** | **Study** | **Nr of patients** | **Management** | **Recovery time** |
| --- | --- | --- | --- | --- |
| Post-procedural pain | Ji  *et al.* (2016) | 8 | Pethidine injections | Not stated |
| Hepatic abscesses | Li *et al.* (2015)^11^ | 2 | Percutaneous drainage | 15-18 days |
|  | Liu *et al.* (2017)^12^ | 2 | Drainage | Not stated |
|  | Reading *et al.* (1988)^16^ | 2 | Surgical drainage (1), not stated (1) | More than four months |
| Mild fever | Sun *et al.* (2015) | 12 | Symptomatic treatment | Not stated |
| Post-embolization syndrome | Reading *et al.* (1988) | 6 | Opiate analgesia | 1-2 days |
| Biloma | Liu *et al.* (2017)^12^ | 2 | Surgical resection (1), drainage followed by right hemihepatectomy (1) | Not stated |
| Ischemic cholecystitis | Bozkaya *et al.* (2014)^5^ | 1 | Antibiotic treatment | 3 days |
| Low haemoglobin | Kirnap *et al.* (2018) | 1 | Two packs of red blood cell suspension | 3 days |
| Transient allergic rash | Özden *et al.* (2017) | 1 | Parenteral steroids | Not stated |
| Lasting pain | Li *et al.* (1997) | Not reported | 1-2 pethidine injection | Not stated |

**References**

1. Lowe LH, Marchant TC, Rivard DC, Scherbel AJ. Vascular malformations: Classification and terminology the radiologist needs to know. Semin Roentgenol. 2012;47(2):106-17.

2. Brown DB, Gould JE, Gervais DA, Goldberg SN, Murthy R, Millward SF, et al. Transcatheter therapy for hepatic malignancy: Standardization of terminology and reporting criteria. J Vasc Interv Radiol. 2009;20(7 Suppl):S425-34.

3. Eisenhauer EA, Therasse P, Bogaerts J, Schwartz LH, Sargent D, Ford R, et al. New response evaluation criteria in solid tumours: Revised recist guideline (version 1.1). Eur J Cancer. 2009;45(2):228-47.

4. Filippiadis DK, Binkert C, Pellerin O, Hoffmann RT, Krajina A, Pereira PL. Cirse quality assurance document and standards for classification of complications: The cirse classification system. Cardiovasc Intervent Radiol. 2017;40(8):1141-6.

5. Bozkaya H, Cinar C, Besir FH, Parildar M, Oran I. Minimally invasive treatment of giant haemangiomas of the liver: Embolisation with bleomycin. Cardiovasc Intervent Radiol. 2014;37(1):101-7.

6. Cao X, He N, Sun J, Wang S, Ji X, Wang J, et al. Interventional treatment of huge hepatic cavernous hemangioma. Chin Med J (Engl). 2000;113(10):927-9.

7. Farges O, Daradkeh S, Bismuth H. Cavernous hemangiomas of the liver: Are there any indications for resection? World J Surg. 1995;19(1):19-24.

8. Firouznia K, Ghanaati H, Alavian SM, Nassiri Toosi M, Ebrahimi Daryani N, Jalali AH, et al. Management of liver hemangioma using trans-catheter arterial embolization. Hepat Mon. 2014;14(12):e25788.

9. Ji J, Gao J, Zhao L, Tu J, Song J, Sun W. Computed tomography-guided radiofrequency ablation following transcatheter arterial embolization in treatment of large hepatic hemangiomas. Medicine (Baltimore). 2016;95(15):e3402.

10. Li GW, Zhao ZR, Li BS, Liu XG, Wang ZL, Liu QF. Source of blood supply and embolization treatment in cavernous hemangioma and sclerosis of the liver. World J Gastroenterol. 1997;3(3):147-9.

11. Li Y, Jia Y, Li S, Wang W, Wang Z, Wang Y, et al. Transarterial chemoembolization of giant liver haemangioma: A multi-center study with 836 cases. Cell Biochem Biophys. 2015;73(2):469-72.

12. Liu X, Yang Z, Tan H, Huang J, Xu L, Liu L, et al. Long-term result of transcatheter arterial embolization for liver hemangioma. Medicine (Baltimore). 2017;96(49):e9029.

13. Martin B, Roche A, Radice L, Aguilar K, Kraiem C. [does arterial embolization have a role in the treatment of cavernous hemangioma of the liver in adults?]. Presse Med. 1986;15(23):1073-6.

14. Ouyang Y, Ouyang XH, Yu M, Gu SB. Frequency of arteriovenous shunts in hepatic cavernous hemangiomas in adults as seen on selective arteriography and postembolization radiography. CardioVascular and Interventional Radiology. 2001;24(3):161-7.

15. Özden İ, Poyanlı A, Önal Y, Demir AA, Hoş G, Acunaş B. Superselective transarterial chemoembolization as an alternative to surgery in symptomatic/enlarging liver hemangiomas. World J Surg. 2017.

16. Reading NG, Forbes A, Nunnerley HB, Williams R. Hepatic haemangioma: A critical review of diagnosis and management. Q J Med. 1988;67(253):431-45.

17. Srivastava DN, Gandhi D, Seith A, Pande GK, Sahni P. Transcatheter arterial embolization in the treatment of symptomatic cavernous hemangiomas of the liver: A prospective study. Abdom Imaging. 2001;26(5):510-4.

18. Sun JH, Nie CH, Zhang YL, Zhou GH, Ai J, Zhou TY, et al. Transcatheter arterial embolization alone for giant hepatic hemangioma. PloS one. 2015;10(8):e0135158.

19. Wang L, Deng C, Li J, Huang X, Fan J, Wang X, et al. Application of a new triple sequential embolization method in treatment of hepatic hemangioma. International Journal of Clinical and Experimental Medicine. 2016;9(2):5255-63.

20. Zeng Q, Li Y, Chen Y, Ouyang Y, He X, Zhang H. Gigantic cavernous hemangioma of the liver treated by intra-arterial embolization with pingyangmycin-lipiodol emulsion: A multi-center study. CardioVascular and Interventional Radiology. 2004;27(5):481-5.

21. Downs SH, Black N. The feasibility of creating a checklist for the assessment of the methodological quality both of randomised and non-randomised studies of health care interventions. J Epidemiol Community Health. 1998;52(6):377-84.

22. Akamatsu N, Sugawara Y, Komagome M, Ishida T, Shin N, Cho N, et al. Giant liver hemangioma resected by trisectorectomy after efficient volume reduction by transcatheter arterial embolization: A case report. J Med Case Rep. 2010;4:283.

23. Althaus S, Ashdown B, Coldwell D, Helton WS, Freeny PC. Transcatheter arterial embolization of two symptomatic giant cavernous hemangiomas of the liver. CardioVascular and Interventional Radiology. 1996;19(5):364-7.

24. Bozkaya H, Cinar C, Ünalp Ö V, Parildar M, Oran I. Unusual treatment of kasabach-merritt syndrome secondary to hepatic hemangioma: Embolization with bleomycin. Wiener klinische Wochenschrift. 2015;127(11-12):488-90.

25. Buffet C, Fritsch J, Etienne JP. Giant hemangiomas of the liver in adults: 3 cases. [french]. Gastroenterol Clin Biol. 1982;6(6-7):531-8.

26. Deutsch GS, Yeh KA, Bates WB, Tannehill WB. Embolization for management of hepatic hemangiomas. Am Surg. 2001;67(2):159-64.

27. Fung EPY, Luk WH, Loke TK, Chan JCS. Kasaback-merritt syndrome treated by transarterial embolisation of giant cavernous haemangioma. Journal of the Hong Kong College of Radiologists. 2003;6(3):162-4.

28. Giavroglou C, Economou H, Ioannidis I. Arterial embolization of giant hepatic hemangiomas. CardioVascular and Interventional Radiology. 2003;26(1):92-6.

29. Igarashi G, Mikami K, Sawada N, Endo T, Sueyoshi N, Sato K, et al. Interventional treatment for giant hepatic hemangioma accompanied by arterio-portal shunt with ascites. Intern Med. 2018.

30. Jin S, Shi XJ, Sun XD, Wang SY, Wang GY. Sclerosing cholangitis secondary to bleomycin-iodinated embolization for liver hemangioma. World J Gastroenterol. 2014;20(46):17680-5.

31. Kapoor S, Pal S, Chattopadhyay TK. Giant hemangioma of the liver presenting with abdominal compartment syndrome: A case report. European Surgery - Acta Chirurgica Austriaca. 2005;37(1):52-4.

32. Koniaris LG, Seibel JA, Geschwind JF, Sitzmann JV. Can ethanol therapies injure the bile ducts? Hepatogastroenterology. 2003;50(49):69-72.

33. Malagari K, Alexopoulou E, Dourakis S, Kelekis A, Hatzimichail K, Sissopoulos A, et al. Transarterial embolization of giant liver hemangiomas associated with kasabach-merritt syndrome: A case report. Acta Radiol. 2007;48(6):608-12.

34. Meguro M, Soejima Y, Taketomi A, Ikegami T, Yamashita Y, Harada N, et al. Living donor liver transplantation in a patient with giant hepatic hemangioma complicated by kasabach-merritt syndrome: Report of a case. Surgery Today. 2008;38(5):463-8.

35. Mohan S, Gupta A, Verma A, Kathura MK, Baijal SS. Case report: Non-surgical management of a giant liver hemangioma. Indian Journal of Radiology and Imaging. 2007;17(2):81-3.

36. Moschouris H, Malagari K, Kornezos I, Papadaki MG, Gkoutzios P, Matsaidonis D. Unenhanced and contrast-enhanced ultrasonography during hepatic transarterial embolization and chemoembolization with drug-eluting beads. CardioVascular and Interventional Radiology. 2010;33(6):1215-22.

37. Nobuoka T, Katsuramaki T, Mizuguchi T, Shima H, Kimura Y, Mukaiya M, et al. A case of giant hepatic hemangioma with spontaneous intratumoral bleeding. Tumor Research. 2004;39:1-5.

38. Pachera S, Nishio H, Yamada H, Yokoyama Y, Ebata T, Igami T, et al. Superextended hepatectomy for resection of multiple giant hemangiomas: Report of a case. Surgery Today. 2009;39(5):452-5.

39. Roche A, Doyon D, Harry G, Weingarten A, Edouard A. [hepatic arterial embolisation. 35 cases (author's transl)]. Nouv Presse Med. 1978;7(8):633-7.

40. Szejnfeld D, Nunes TF, Fornazari VA, de Matos CA, Gonzalez AM, D'Ippolito G, et al. Transcatheter arterial embolization for unresectable symptomatic giant hepatic hemangiomas: Single-center experience using a lipiodol-ethanol mixture. Radiol Bras. 2015;48(3):154-7.

41. Tanaka A, Morimoto T, Yamamori T, Moriyasu F, Yamaoka Y. Atypical liver hemangioma with shunt: Long-term follow-up. J Hepatobiliary Pancreat Surg. 2002;9(6):750-4.

42. Tarazov PG, Polysalov VN, Ryzhkov VK. Hemangiomatosis of the liver and spleen: Successful treatment with embolization and splenectomy. AJR Am J Roentgenol. 1990;155(6):1235-6.

43. Tarazov PG, Polysalov VN. Arterial embolization in hepatic cavernous haemangioma complicated by anaemia. Journal of Interventional Radiology. 1993;8(2):71-3.

44. Xu LN, Huang ZQ. Resection of hepatic caudate lobe hemangioma: Experience with 11 patients. Hepatobiliary Pancreat Dis Int. 2010;9(5):487-91.

45. Yang Z, Tan H, Liu X, Sun Y. Extremely giant liver hemangioma (50 cm) with kasabach-merritt syndrome. J Gastrointest Surg. 2017;21(10):1748-9.

46. Zhou JX, Huang JW, Wu H, Zeng Y. Successful liver resection in a giant hemangioma with intestinal obstruction after embolization. World J Gastroenterol. 2013;19(19):2974-8.
